# Cross-sectional and longitudinal associations of circulating omega-3 and omega-6 fatty acids with lipoprotein particle concentrations and sizes: population-based cohort study with 6-year follow-up

**DOI:** 10.1186/1476-511X-13-28

**Published:** 2014-02-07

**Authors:** Pekka Mäntyselkä, Leo Niskanen, Hannu Kautiainen, Juha Saltevo, Peter Würtz, Pasi Soininen, Antti J Kangas, Mika Ala-Korpela, Mauno Vanhala

**Affiliations:** 1 Faculty of Health Sciences, School of Medicine, University of Eastern Finland, Kuopio 70211, Finland; 2 Primary Health Care Unit, Kuopio University Hospital, Kuopio 70211, Finland; 3 Finnish Medicines Agency Fimea, Helsinki 00280, Finland; 4 Unit of Primary Health Care, Helsinki University Central Hospital, Helsinki 00014, Finland; 5 Department of General Practice, University of Helsinki, Helsinki 00014, Finland; 6 Department of Medicine, Central Finland Central Hospital, Jyväskylä 40620, Finland; 7 Institute for Molecular Medicine Finland, University of Helsinki, Helsinki 00014, Finland; 8 Computational Medicine, Institute of Health Sciences, University of Oulu, Oulu 90014, Finland; 9 NMR Metabolomics Laboratory, School of Pharmacy, University of Eastern Finland, Kuopio 70211, Finland; 10 Computational Medicine, School of Social and Community Medicine and the Medical Research Council Integrative Epidemiology Unit, University of Bristol, Bristol, UK; 11 Oulu University Hospital, Oulu 90014, Finland; 12 Primary Health Care Unit, Central Finland Central Hospital, Jyväskylä 40620, Finland

**Keywords:** Lipoprotein profile, Fatty acid, Cohort study

## Abstract

**Background:**

Cross-sectional studies have suggested that serum omega-3 (n-3) and omega-6 (n-6) polyunsaturated fatty acids (PUFAs) are related to favorable lipoprotein particle concentrations. We explored the associations of serum n-3 and n-6 PUFAs with lipoprotein particle concentrations and sizes in a general population cohort at baseline and after 6 years.

**Findings:**

The cohort included 665 adults (274 men) with a 6-year follow-up. Nutritional counseling was given at baseline. Serum n-3 and n-6 PUFAs and lipoprotein particle concentrations and the mean particle sizes of VLDL, LDL, and HDL were quantified by nuclear magnetic resonance (NMR) spectroscopy for all baseline and follow-up samples at the same time. Concentrations of n-3 and n-6 PUFAs were expressed relative to total fatty acids. At baseline, n-3 PUFAs were not associated with lipoprotein particle concentrations. A weak negative association was observed for VLDL (P = 0.021) and positive for HDL (P = 0.011) particle size. n-6 PUFA was negatively associated with VLDL particle concentration and positively with LDL (P < 0.001) and HDL particle size (P < 0.001). The 6-year change in n-3 PUFA correlated positively with the change in particle size for HDL and LDL lipoproteins but negatively with VLDL particle size. An increase in 6-year levels of n-6 PUFAs was negatively correlated with the change in VLDL particle concentration and size, and positively with LDL particle size.

**Conclusion:**

Change in circulating levels of both n-3 and n-6 PUFAs, relative to total fatty acids, during 6 years of follow-up are associated with changes in lipoprotein particle size and concentrations at the population level.

## Introduction

Circulating polyunsaturated fatty acids (PUFAs) may reflect dietary PUFAs although they may also be influenced by other factors such as obesity and insulin resistance. There is observational evidence that dietary intake of omega-3 (n-3) PUFA is associated with a favorable lipoprotein profile especially with higher concentrations of HDL-particles and larger HDL particle size but also with lower concentrations of LDL particles and larger LDL particle size and decreasing concentration of VLDL particles and smaller VLDL particles [1,2]. However, daily treatment with n-3 fatty acids has not convincingly been associated with reduced cardiovascular morbidity and mortality [3,4]. It has been suggested that blood level of n-3 PUFAs may be a better indicator of cardiovascular risk than n-3 supplementation [5]. Omega-6 (n-6) PUFAs may reduce inflammation, insulin resistance and decreases the risk of coronary heart disease (CHD) [6] but the clinical benefits of n-6 PUFAs related to decreasing cardiovascular diseases and mortality remains to established [7].

Motoyama et al. have shown that circulating serum n-6 and n-3 PUFAs are inversely related to triglycerides in middle aged men in several cross-sectional studies [8]. Higher serum concentrations of n-6 PUFAs are further associated with lower concentrations of LDL particles and large VLDL particles and a higher concentration of large HDL particles [9]. However, no study has assessed the longitudinal changes of serum n-3 and n-6 PUFAs and their associations with changes in lipoprotein particle concentrations and sizes at the population level. We therefore analyzed the longitudinal associations between changes in serum n-3 and n-6 PUFA concentrations with corresponding changes of lipoprotein particle concentrations and sizes in a population-based setting during 6-year follow-up.

## Methods

### Study population

All of the inhabitants in the town of Pieksämäki, Finland, who were born in 1942, 1947, 1952, 1957, and 1962 (n = 1,294) were invited for a health check-up in 1997-1998 (baseline visit) and again in 2003-2004 At the Primary Health Care Unit in Pieksämäki, Finland. Of those invited, 923 (71%) participated in the first check-up [10]. Of these, 688 subjects (74.5%) attended the second health check-up six years (mean follow-up period) later. All variables analysed in the present study were available from 665 subjects (274 men and 391 women). During the first health examination, the nurses were instructed to counsel all the participants to embrace more healthy lifestyles, including advising them to replace hard fats (e.g. butter, greasy milk, greasy red meat) with smooth fats (e.g. vegetable oil, chicken, fish) and liquid margarines, use olive oil or rapeseed oil in preparing food, and increase the consumption of fish. The protocol was approved by the Ethics Committee of Kuopio University Hospital. All of the participants gave their informed written consent.

### Clinical and laboratory procedures

Both health check-ups were performed by the same two nurses. Waist circumference was measured and body mass index (BMI) was calculated. Blood samples were taken after an overnight fast. Glucose and serum lipids were measured by standard enzymatic methods [10] from fresh serum samples and analyzed in the laboratory of the Kuopio University Hospital. Insulin was determined by Phadeseph Insulin Radio Immunoassay (RIA) 100 method (Pharmacia Diagnostics AB, Uppsala, Sweden). Concentrations of lipoprotein subclass particles were analysed by high-throughput nuclear magnetic resonance (NMR) spectroscopy of native serum samples as described previously [11-14]. The mean size of the VLDL, LDL, and HDL particles was calculated by weighting the corresponding subclass diameters with their particle concentrations. The diversity of fatty acid saturation was quantified from serum extract samples [13]. NMR spectroscopy was used conducted for all baseline and follow-up samples at the same time in 2009. For statistical analysis, the ratios of PUFA concentrations (mmol/L) to total fatty acid concentration (mmol/L) were examined.

### Statistical methods

The data are presented as means with standard deviations. The 95% confidence intervals for the lipoprotein particle concentrations and sizes were obtained by bias-corrected, accelerated bootstrapping (5000 replications). Statistical significance for hypotheses of linearity was evaluated by analysis of variance (ANOVA), Jonckheere-Terpstra test, or Cochran-Armitage test. Linearity across the particle concentrations of tertiles of n-3 and n-6 PUFAs was tested by using bootstrap-type general linear models with an appropriate contrast adjusted for age, sex, smoking and BMI, antihyperglycemic medication and lipid-lowering medication at baseline. Respectively, associations between the change in n-3 and n-6 PUFAs% with changes in total VLDL, LDL, and HDL particle concentrations and mean particle sizes during follow-up were estimated with regression analysis using Sidak-adjusted probabilities. These longitudinal changes were adjusted for age, sex, smoking and BMI, antihyperglycemic medication and lipid-lowering medication at baseline. Repeated data (relative change over time) were analysed using generalising estimating equations (GEE) models with the unstructured correlation structure. Generalized estimating equations were developed as an extension of the general linear model (eg, OLS regression analysis) to analyze longitudinal and other correlated data.

## Results

Table 1 shows the baseline characteristics of the study population by gender. The mean age was 46 years and BMI 26.5 kg/m^2^. The use of lipid lowering, antihypertensive and glucose lowering agents was not frequent in the study population at baseline.

**Table 1:** Baseline characteristics of the study population

	**Women**	**Men**	**P-value**
	**N = 391**	**N = 274**	
Age, years, mean (SD)	46 (6)	46 (6)	0.52
Body mass index, kg/m^2^, mean (SD)	26.3 (5.2)	26.8 (3.6)	0.21
Waist, cm, mean (SD)	83 (23)	94 (10)	<0.001
Lipid lowering medication, n (%)	5 (1)	11 (4)	0.023
Antihypertensive medication, n (%)	36 (9)	33 (12)	0.24
Antihyperglycemic medication, n (%)	1 (0)	6 (2)	0.022
Total cholesterol, mmol/L, mean (SD)	5.6 (0.97)	5.7 (0.98)	0.047
HDL cholesterol, mmol/L, mean (SD)	1.5 (0.33)	1.3 (0.30)	<0.001
Triglycerides, mmol/L, mean (SD)	1.2 (0.63)	1.6 (0.92)	<0.001
Glucose, mmol/L, mean (SD)	5.6 (0.59)	5.9 (0.96)	<0.001
Insulin, mU/L, mean (SD)	9.9 (7.3)	10.3 (5.2)	0.38
Current smoking, n (%)	80 (21)	77 (27)	0.024
Reported use of alcohol, n (%)	309 (79)	239 (88)	0.004

Table 2 shows the mean particle concentrations and the particle sizes of VLDL, LDL, and HDL across tertiles of n-3 and n-6 PUFA concentrations, relative to total fatty acids. No significant associations were observed between n-3 PUFAs and concentrations of lipoprotein particles. However, serum n-3 PUFAs was negatively associated with VLDL particle size (P = 0.021) and positively with HDL particle size (P = 0.011). There was a strong negative relationship between n-6 PUFAs and VLDL particle concentration whereas n-6 PUFAs did not associate with LDL and HDL particle concentrations. There was a negative linear trend for VLDL particle size and positive linear trend for LDL and HDL particle size across n-6 PUFA relative concentrations. Compared to n-3 PUFAs, the relationships between n-6 PUFAs and lipoprotein particle sizes were stronger (all P < 0.001).

**Table 2:** Baseline VLDL, LDL and HDL particle concentrations and sizes according to n-3 PUFA and n-6 PUFA tertiles

	**Tertiles of N-3 PUFAs**			
	**(Percentage of total fatty acid concentration)**			
	**I**	**II**	**III**	
	**<3.16%**	**3.16% to 3.87%**	**>3.87%**	P for linearity*
VLDL, mean (SD)				
Concentration, nmol/L	84.8 (26.7)	93.0 (30.4)	87.1 (30.4)	0.81
Mean size, nm	36.4 (1.31)	36.5 (1.39)	36.1 (1.21)	0.021
LDL, mean (SD)				
Concentration, nmol/L	543 (119)	563 (122)	557 (121)	0.91
Mean size, nm	23.5 (0.17)	23.5 (0.18)	23.5 (0.15)	0.084
HDL, mean (SD)				
Concentration, μmol/L	7.25 (0.79)	7.36 (0.82)	7.46 (0.79)	0.086
Mean size, nm	9.92 (0.23)	9.93 (0.24)	9.98 (0.24)	0.011
	**Tertiles of N-6 PUFAs**			
	**I**	**II**	**III**	
	**<32.7%**	**32.7% to 36.1%**	**>36.1%**	P for linearity*
VLDL, mean (SD)				
Concentration, nmol/L	110 (29.3)	82.5 (22.6)	72.3 (20.7)	<0.001
Mean size, nm	37.4 (1.32)	36.1 (0.99)	35.6 (0.81)	<0.001
LDL, mean (SD)				
Concentration, nmol/L	589 (122)	537 (117)	537 (116)	0.15
Mean size, nm	23.5 (0.19)	23.6 (0.14)	23.6 (0.13)	<0.001
HDL, mean (SD)				
Concentration, μmol/L	7.28 (0.84)	7.35 (0.82)	7.44 (0.75)	0.28
Mean size, nm	9.85 (0.20)	9.97 (0.23)	10.0 (0.25)	<0.001

*adjusted for age, sex, smoking, BMI and antihyperglycemic medication and lipid-lowering medication at baseline.

In general, VLDL particle concentration and size increased (P < 0.001) and HDL particle concentration decreased (P < 0.001) whereas no significant changes were found for LDL particle concentrations and for LDL and HDL particle sizes (Additional file 1: Figure S1). Mean change of triglycerides (men and women together) was -0.05 mmol/L (95% CI: -0.11 to 0.01 mmol/L). Table 3 shows the partial correlations of 6-year changes in serum n-3 and n-6 (in% of total fatty acids) with changes in VLDL, LDL, and HDL lipoprotein particle concentrations and mean particle sizes. The change in serum n-3 PUFAs during follow-up correlated positively with the change in LDL particle size (r = 0.13) and HDL particle size (r = 0.12) although the correlations were weak. Modest inverse correlation (r = -0.20) was found between the changes of n-3 PUFAs and VLDL particle size. The change of n-3 PUFAs was not associated with changes in VLDL, LDL and HDL particle concentrations during follow-up (P > 0.05 for all).

**Table 3:** Partial correlations between changes in n-3 and n-6 PUFAs (in % of total fatty acids) and changes in lipoprotein particle concentrations and mean sizes

	**Change of**	
	**n-3 PUFAs**	**n-6 PUFAs**
	**r (95% CI)**^**#**^	**r (95% CI)**^**#**^
Change of		
VLDL particle concentration	-0.05 (-0.12 to 0.02)	-0.37 (-0.44 to -0.30)***
VLDL particle mean size	-0.20 (-0.28 to -0.12)***	-0.55 (-0.61 to -0.48)***
LDL particle concentration	-0.02 (-0.07 to 0.05)	0.00 (-0.06 to 0.06)
LDL particle mean size	0.13 (0.06 to 0.20)**	0.34 (0.26 to 0.42)***
HDL particle concentration	0.09 (0.00 to 0.18)	-0.08 (-0.16 to 0.02)
HDL particle mean size	0.12 (0.04 to 0.20)*	0.05 (-0.03 to 0.14)

^#^The 95% confidence intervals were obtained by bias-corrected bootstrapping (5,000 replications).

Sidak-adjusted probabilities. Adjusted for age, sex, smoking, BMI and antihyperglycemic medication and lipid-lowering medication at baseline.

*<0.05 **<0.01 ***<0.001.

The change in n-6 PUFAs correlated negatively with the change in VLDL particle concentration (r = -0.37) and VLDL particle size (r = -0.55); the correlation with change in LDL particle size was positive (r = 0.34). The change in n-6 PUFAs was not significantly correlated with the change in HDL particle concentration and size or with change in LDL particle concentration.

## Discussion

The present population-based study comprising 665 middle-aged subjects showed that serum levels of both n-3 and n-6 PUFA, relative to total fatty acids, were associated with lipoprotein particle concentrations and sizes. Longitudinal changes in these fatty acid proportions paralleled various changes in lipoprotein particle numbers and sizes. This appears to be the first study showing longitudinal association between changes in serum PUFA levels and lipoprotein particle concentrations in a population consuming their habitual diet. The baseline results of the present study are in line with the previous cross-sectional study of 1098 men conducted by Choo et al. [9].

Previously, Motoyama et al. found in the population-based large study that n-6 and n-3 PUFAs were inversely related to serum triglycerides which are carried by VLDL [8]. VLDL lipoprotein has been found to associate with increased cardiovascular risk [15]. In line with the previous study by Choo et al. [9] higher n-6 PUFA concentration at baseline associated with smaller VLDL particle size and concentration in the present study. However there was not a clear association between n-3 PUFAs and VLDL particles at baseline. In addition, the strongest longitudinal correlations were found for the negative associations between n-6 PUFAs and VLDL particle concentration and size. Small low-density lipoprotein particles have been shown to be a cardiovascular risk factor predicting coronary artery disease and events [16-18]. In the present study, there was not a difference in LDL particle sizes between n-3 tertiles at baseline and the difference between n-6 PUFA tertiles was only modest. However, the present findings suggest that the change of PUFA levels, especially n-6 PUFAs, associate positively with the change of LDL particle size. Increased circulating levels of n-3 and n-6 PUFAs may contribute to favorable non-HDL (LDL and VLDL) particle changes.

Serum PUFA concentrations showed an association with mean HDL particle size at baseline. In addition, the change of n-3 PUFAs correlated with the change in HDL particle size. The FinnTwin-study suggested that a higher consumption of n-3 PUFA was related to changes in HDL subspecies toward a larger mean particle size in men [1]. That association remained significant in analyses controlling for genetic and environmental influences and for confounding factors like BMI, smoking and physical activity. Here we were able to control for additional potential confounding factors which yielded consistent findings with the FinnTwin-study.

The serum concentrations of fatty acids are generally regarded as markers of dietary intake although adiposity and genetics may contribute [19]. We did not assess the macronutrient dietary intake from this population. In the population-based Kuopio Ischemic Heart Disease (KIHD) study dietary intake of PUFAs correlated with serum PUFAs (r = 0.50) [20]. The KIHD study was performed in an area in close proximity of this study area and subjects in both KIHD and in this study share similar socio-demographic characteristics. The Cardiovascular Risk in Young Finns Study provided consistent findings about the relationship between dietary intake and serum levels of PUFAs as quantified by NMR spectroscopy [21]. We assume that serum PUFA levels in the present study primarily reflect dietary intake of PUFAs. It is possible that most pronounced change in the dietary habits was the change in fat quality. In the lake area of this study population, the potential increased intake of n-3 PUFAs was most likely from the lean white fish [22]. However, these assumptions cannot be confirmed based on these data because we did not have the measurement of diet.

The observational nature of this study, with only two health checkups and missing dietary intake information may be taken as a limitation. However, the present study represents a general population with several years of follow-up and with measurements of the circulating lipoprotein particle profile as well as PUFAs conducted for all baseline and follow-up samples at the same time.

In conclusion, at the population level, the 6-year relative increases in serum levels of both n-3 and n-6 PUFAs are associated with concurrent favorable changes in the lipoprotein particle profile among men and women with a mean age of 46 years.

## Abbreviations

CHD: Coronary heart disease; HDL: High density lipoprotein; n-3: Omega-3; n-6: Omega-6; NMR: Nuclear magnetic resonance; PUFA: Poly-unsaturated fatty acid; LDL: Low density lipoprotein; VLDL: Very low density lipoprotein.

## Competing interests

The authors declare that they have no competing interests.

## Authors’ contributions

MV was responsible for the collection of data. PM, MV and HK were responsible for study concept and design. LN, PM, HK, JS, PW, MAK and MV were responsible for data interpretation. The NMR analyses were conducted by MAK, PS and AK. The manuscript was drafted PM and LN. All authors critically revised the manuscript for important intellectual content and approved the final version of manuscript.

## Supplementary Material

Additional file 1: Figure S1 Relative change over time of 6 years (from baseline to follow-up) of VLDL, LDL and HDL lipoprotein concentrations and sizes adjusted for age, sex, smoking, BMI and antihyperglycemic medication and lipid-lowering medication at baseline. Figure representing total changes of main outcome measures. Click here for file
