# Supplementary material for: Cross-sectional and longitudinal associations of circulating omega-3 and omega-6 fatty acids with lipoprotein particle concentrations and sizes: population-based cohort study with 6-year follow-up
Source: Lipids Health Dis. 2014 Feb 7;13:28. doi: 10.1186/1476-511X-13-28 (PMC3922432; doi:10.1186/1476-511X-13-28)
Supplement: Supplementary file 1 [file 1476-511X-13-28-S1.docx]

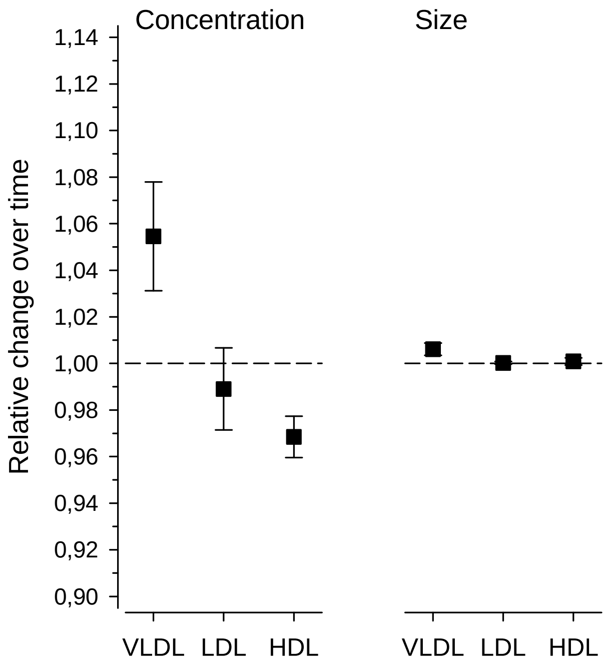
Concentration
Size
Relative change over time
1,14
1,12
1,10
1,08
1,06
1,04
1,02
1,00
0,98
0,96
0,94
0,92
0,90
VLDL
LDL
HDL
VLDL
LDL
HDL
